# Rapid Sampling of *Escherichia coli* After Changing Oxygen Conditions Reveals Transcriptional Dynamics

**DOI:** 10.3390/genes8030090

**Published:** 2017-02-28

**Authors:** Joachim von Wulffen, Andreas Ulmer, Günter Jäger, Oliver Sawodny, Ronny Feuer

**Affiliations:** 1 Institute for System Dynamics, University of Stuttgart, Keplerstraße 7, 70174 Stuttgart, Germany; vonwulffen@isys.uni-stuttgart.de (J.W.); ulmer.bio@gmail.com (A.U.); ronny.feuer@isys.uni-stuttgart.de (R.F.); 2 Insitute of Medical Genetics and Applied Genomics, University of Tübingen, Geschwister-Scholl-Platz, 72074 Tübingen, Germany; guenter.jaeger@cegat.de

**Keywords:** transcriptional elongation rates, RNA sequencing, transition, oxygen, *Escherichia coli*

## Abstract

*Escherichia coli* is able to shift between anaerobic and aerobic metabolism by adapting its gene expression, e.g., of metabolic genes, to the new environment. The dynamics of gene expression that result from environmental shifts are limited, amongst others, by the time needed for regulation and transcription elongation. In this study, we examined gene expression dynamics after an anaerobic-to-aerobic shift on a short time scale (0.5, 1, 2, 5, and 10 min) by RNA sequencing with emphasis on delay times and transcriptional elongation rates (TER). Transient expression patterns and timing of differential expression, characterized by delay and elongation, were identified as key features of the dataset. Gene ontology enrichment analysis revealed early upregulation of respiratory and iron-related gene sets. We inferred specific TERs of 89 operons with a mean TER of 42.0 nt/s and mean delay time of 22.4 s. TERs correlate with sequence features, such as codon bias, whereas delay times correlate with the involvement of regulators. The presented data illustrate that at very short times after a shift in oxygenation, extensional changes of the transcriptome, such as temporary responses, can be observed. Besides regulation, TERs contribute to the dynamics of gene expression.

## 1. Introduction

*Escherichia coli* is able to adapt to various changes in environmental conditions. An important determinant of growth is the presence of extracellular oxygen. Oxygen availability changes in the natural niches of *E. coli*, as well as in biotechnological settings. In industrial scale continuously stirred tank reactors (CSTR) inhomogeneities of available oxygen arise from insufficient mixing compared to the laboratory scale. Mixing times in large CSTRs are in the range of 2 min or more (140 s for a 120,000-L reactor [1]) while oxygen depletion can occur in less than a minute [2]. Therefore, cells circulating through the CSTR volume experience a constantly changing oxygen supply, necessitating constant adaptation of the cells to the respective conditions. Inhomogeneities of the dissolved oxygen are presumed to be an important cause for yield losses after upscaling [2,3]. In this study, we analyze the early transcriptional responses of an anaerobic *E. coli* batch culture to the onset of aeration by RNA sequencing.

Previous studies on transcriptional responses to an aerobic shift (from anaerobic to aerobic conditions) considered chemostat cultivations and longer time scales only (5–60 min [4], 2–20 min [5], and 5–120 min [6]), or only moderate shifts in oxygen supply [6,7]. These studies were mostly motivated by the naturally occurring transition between anaerobic (in the host’s intestines) and aerobic (outside the host) conditions. Here, we focus on fast transcriptomic changes of a batch culture in response to an aerobic shift using RNA sequencing.

Although other substrates are also inhomogeneously distributed in large scale cultivations, oxygen has a crucial role. Firstly, oxygen is not a prerequisite for growth, as *E. coli* cells grow anaerobically as well, but with a lower rate. However, switching from anaerobic to aerobic metabolism requires extensive adjustments of the cellular proteome and, as a precondition, of the transcriptome. This might even include the expression of genes that code for suboptimally efficient enzymes if their expression is faster than their more efficient alternatives [8]. Secondly, exposure to oxygen can cause excessive generation of reactive oxygen species (ROS), which provoke cellular stress. On the transcriptomic level, expression of stress genes can be seen indicative of present stress conditions [9].

Gene expression is, in terms of energy consumption, a costly undertaking considering limited cellular resources, as demonstrated in a setting of fluctuating carbon and nitrogen supply [10,11]. Therefore, tight regulation is needed to avoid unnecessary gene expression and assure optimal adaptation considering evolutionary pressure [12].

Another aspect demonstrating the need for basal expression of yet unutilized genes is the response time elapsing from the occurrence of the environmental shift to increased levels of functional protein. There are several phases that need to pass before the newly synthesized protein becomes active, including sensing and regulation of transcriptional initiation (delay time), transcription elongation, and translation, as well as post-translational modifications. The first two of these, delay time and transcription, can be approached with time series RNA sequencing measurements. Using rifampicin, an inhibitor of transcriptional initiation, operon-specific transcriptional elongation rates (TERs) have been inferred [13]. Using oxygen shift as a trigger for gene expression changes, we were able to derive delay times and TERs and compare them with literature values.

Translation in *E. coli* can occur cotranscriptionally [14], and it has been postulated that ribosomes and RNA polymerases cooperatively determine elongation rates [15,16] and suppress termination (antitermination [17,18,19]). Therefore, TERs are valuable for estimating the minimal amount of time required to synthesize new proteins after environmental shift.

## 2. Materials and Methods

### 2.1. Strain and Growth Conditions

*E. coli* K-12 strain W3110 [20,21] was grown anaerobically in defined medium (Table S1) at pH7 and 37 °C stirred at 500 rpm with a Rushton turbine in a 3-liter bioreactor until the culture reached an optical density at 600 nm (OD_600 nm_) of 3. At that point, the first sample was drawn and air supply was initiated subsequently at 1 L/min. Additional samples were taken at 0.5, 1, 2, 5, and 10 min after the onset of aeration and treated immediately with RNAprotect Cell Reagent (Qiagen, Hilden, Germany) according to manufacturer’s instructions. Cell pellets were stored at −80 °C until preparation. The experiment was repeated in three biological replicates.

### 2.2. RNA Sequencing

Ribosomal RNA was removed from transcripts of RNA protected samples using RiboZero rRNA Removal Kit (Illumina, San Diego, CA, USA). Sequencing libraries were prepared with TruSeq RNA Sample Preparation Kit v2 (Illumina). The quality of RNA preparations was assessed electrophoretically with Agilent BioAnalyzer (Agilent, Santa Clara, CA, USA). Equimolar amounts of all libraries were pooled and loaded onto an Illumina GAIIx flow cell and bound molecules were clonally amplified on a cBot instrument (Illumina). Subsequently, RNA fragments were sequenced on an Illumina HiSeq 2500. The quality of the sequencing was assessed using FastQC [22].

Obtained sequences were aligned against the reference genome NC007779.1 [23] using the tool STAR [24]. Gene counting was performed with HTSeq [25], filtering out overlapping regions. The obtained data have been deposited in the National Center for Biotechnology Information (NCBI)’s Gene Expression Omnibus (GEO) [26] and are accessible through GEO Series accession number GSE71562. The Bioconductor package edgeR [27,28] was applied for statistical analysis. Total mapped read counts ranged from 5.6 million to 11.0 million. Read counts were transformed to counts per million taking into account the different library sizes. Normalization with the respective gene lengths yields reads per kilobase per million reads (RPKM), which are used for plotting. A negative binomial model was fitted with the count data and the common and tag-wise dispersions were estimated. Subsequently, a generalized linear model was fitted to the data and *p* values were calculated. The resulting *p* values were corrected for multiple testing using the false discovery rate (FDR) method [29] (data available in Table S2). Genes with FDR corrected *p* values of less than 0.05 and absolute fold changes greater than 2 were assumed as differentially expressed (DE). Venn diagrams were generated with the VennDiagram R package [30].

### 2.3. Principal Component Analysis

The regularized logarithm transformation of the Bioconductor package DESeq2 was applied to the count data to make the data more homoscedastic [31]. This approach balances the high counts of highly expressed genes by taking the logarithm with the large fold changes (FC) of weakly expressed genes by shrinking. These transformed count numbers were used for principal component analysis (PCA) employing the prcomp() command in R [28,32].

### 2.4. Regulon and Gene Ontology Enrichments

For each time point, genes with an absolute fold change greater than 2 and FDR-corrected *p* values of less than 0.05 were filtered and sorted for up- or downregulation. Enrichment of regulons and gene ontology (GO) classes (biological process) were analyzed using the smart tables functions of Pathway Tools [33,34] with Bonferroni correction.

### 2.5. TER Estimation

As in [13], operon sequences were partitioned into 300-nt bins and counts were generated for each bin using the R package GenomicAlignments [35]. RPKM values were derived from counts as described above and subsequently fitted to the differential equation

$$\frac{d\;[mRNA_{i}]}{d\;t}=s_{b,i}+\Theta \left(t-T_{d,i}\right)\cdot s_{a,i}-\left(\mu +\gamma_{i}\right)\cdot \left[mRNA_{i}\right]\left(t\right)$$

with square brackets denoting concentrations in RPKM, *s_b,i_* basal expression rate, *s_a,i_* activated expression rate, *T_d,i_* the delay time until differential expression sets in, *μ* the growth rate and *γ_i_* degradation rate of the mRNA of gene *i*. The number of measurements included in each bin’s regression had to be adjusted manually in order to estimate the initial adaptation with one change of expression rates, only (i.e., from *s_b,i_* to (*s_b,i_ + s_a,i_*) or from (*s_b,i_ + s_a,i_*) to *s_b,i_*). For example, the *cydAB* operon, illustrated in Figure 5a,b, is transiently upregulated. For the bins 0 to 4, only the first three time points were included in the parameter fit, since expression increases and decreases fast. Parameter estimation of the subsequent bins 5–8 was performed on the initial four time points, since the increased expression was delayed. Where possible, *γ_i_* was taken from the literature [36], if not it was fitted to the data. Θ is a step function and is defined for cases with increasing expression as:

$$\Theta_{incr}\left(t\right)=\left\{\begin{array}{cc} 0 & t<0\\ 1 & t\geq 0 \end{array}\right.$$

and in cases with decreasing expression as $\Theta_{decr}\left(t\right)=1-\Theta_{incr}\left(t\right)$. Numerical parameter estimation was performed using Mathematica 10 (Wolfram, Oxfordshire, UK) using the simulated annealing algorithm [37] with the least squares estimator. Linear regression was applied to estimate the slope of the delay times *T_d_* over the mean bin positions weighted by the 95% confidence intervals of the *T_d_*. The inverse of the estimated slope represents the average TER. The intersection of the regression curve with the 0 nt position of the operon gives the overall delay time of the operon (illustrated in Figure 5a,b).

The codon adaptation index (CAI) was calculated from the operon sequences as described in [38] using the codon frequency table from the Kazusa Codon Usage Database [39]. The index of translation elongation (ITE) was calculated as stated in [40] using the provided data set. Folding energies were calculated from sequences using the ViennaRNA package [41,42]. The regulatory and sigma factor networks were taken from the EcoCyc *E. coli* Database [43].

## 3. Results

*E. coli* K-12 W3110 was cultured anaerobically in a laboratory-scale CSTR. At a predefined point, aeration was initiated. Subsequently, samples were taken within 10 min after the onset of aeration (a time course of the dissolved oxygen concentration is provided in Figure S1). The mRNA of all samples were sequenced and evaluated statistically (Table S2, raw data are available from NCBI GEO, accession number GSE71562).

### 3.1. General Properties of the Dataset

#### 3.1.1. Differentially Expressed Genes

**Figure 1:**
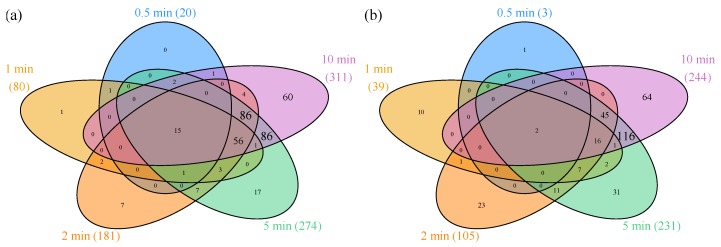
Venn diagrams of differentially expressed (DE) genes. Number of genes with increasing (**a**) and decreasing (**b**) expression compared to anaerobic control at the specified time points (total number of DE genes at the specified time points are provided in brackets). The numbers in overlapping regions represent DE genes at the corresponding time points. Areas are not drawn to scale. Genes are considered DE compared to anaerobic steady state, if |fold change (FC)| > 2 and false discovery rate (FDR) corrected *p* value < 0.05.

Figure 1 shows the number of statistically significant up- and downregulated genes for each time point. Genes with absolute FC greater than 2 and FDR corrected *p* values lower than 0.05 were considered DE. The maximal difference is reached at 10 min where 7.2% of the genome is upregulated and 5.7% downregulated.

The total number of genes that are DE at any time point between 0 and 10 min is 678, out of 4295 mapped genes. This number exceeds the number of DE genes at 10 min (555 genes), so a set of genes exists that is transiently up- or downregulated. Genes that exert significant transient expression patterns lie outside the 10-min ellipses of the Venn diagrams (Figure 1).

In order to get an overview of the transient pattern of differential expression, a heatmap of logFC values was generated (Figure S2). Genes that are DE in at least one timepoint are depicted. Genes are grouped according to transient up- or downregulation and subsequently sorted according to the FC in the 10 min sample. The heatmap indicates that almost half of the genes with differential expression exhibit a transient expression pattern (310 out of 678 genes or 45.7%; areas 2 and 3 in Figure S2).

#### 3.1.2. Principal Component Analysis

**Figure 2:**
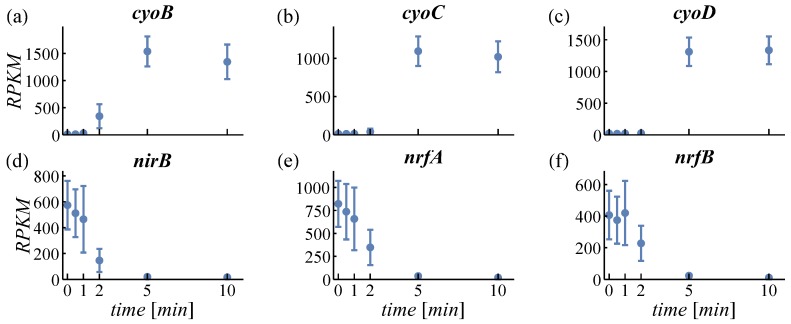
Loadings of principal component 1. Most positively loaded genes are given in the top row (**a**–**c**), negatively loaded genes in the bottom row (**d**–**f**). RPKM: reads per kilobase per million reads.

**Figure 3:**
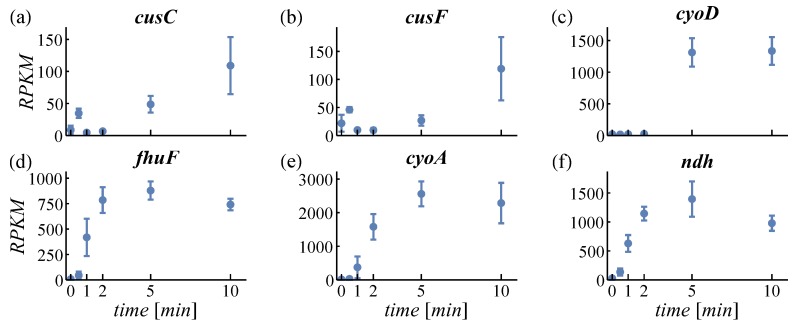
Loadings of principal component 2. Most positively loaded genes are given in the top row (**a**–**c**), negatively loaded genes in the bottom row (**d**–**f**).

PCA is used to structure large datasets and to find the most characteristic expression patterns amongst genes. For PCA, regularized logarithm transformed counts were used in order not to overestimate changes in very high or low expressed genes [31]. The first two principal components cover about 74% of the variance in the data (see scree plot in Figure S3). The loadings of PC1 and PC2 are plotted in Figure S4 (genes can be identified by pointing at the dots). The time courses of the three most positive and negative genes of PC1 and PC2 are provided in Figure 2 and Figure 3, respectively. The genes shown in Figure 2a–c have a positive loading in PC1. Expression of these genes is increasing with time. Figure 2d–f shows genes with negative loading and here expression decreases. Therefore, the feature increasing or decreasing expression is identified by the PCA describing most variance (63%). For PC2, all displayed genes (Figure 3a–c with positive loading, d–f with negative loading) exert an increasing expression. However, positively loaded genes are increasing predominantly between 2 min and 10 min, while negatively loaded genes increase between 0.5 min and 2 min. This feature—early or late responding genes—is the second important property detected by PCA (11% variance).

#### 3.1.3. Regulon and Gene Ontology Enrichments

**Figure 4:**
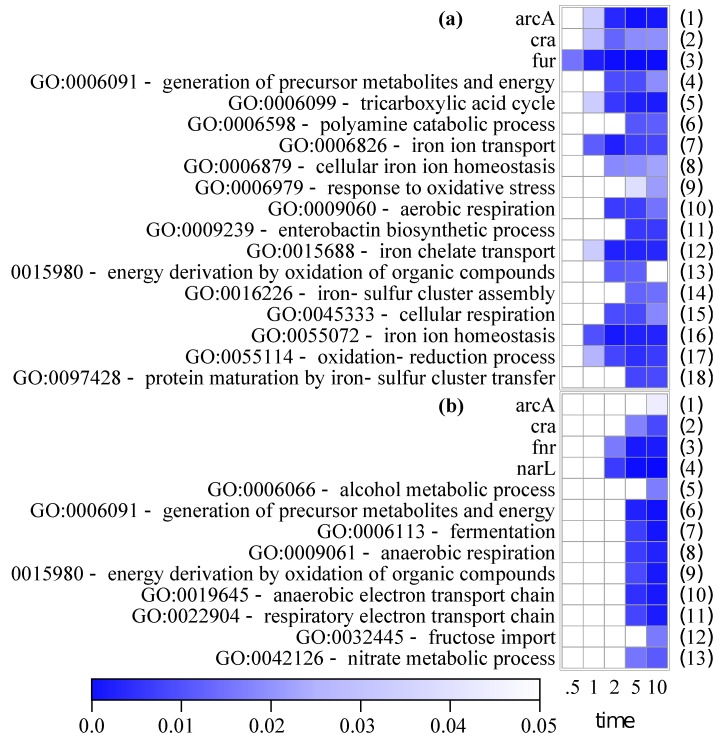
Selection of regulon and gene ontology (GO) enrichments of upregulated (**a**) and downregulated (**b**) genes compared to anaerobic state. Full list is available from Figure S5. *p* values with Bonferroni correction of the enrichments are represented.

To classify the up- and downregulated genes on the functional level, a regulon and GO enrichment analysis was carried out using Pathway Tools [33,34]. Significant enrichments of a selection of regulators and GO terms are plotted in Figure 4a for upregulated genes and in Figure 4b for downregulated genes (the full list of enrichments is available from Figure S5 and Table S3 detailing the matched genes).

The ferric-uptake regulator Fur is the only enriched class at 0.5 min. Its targets remain enriched for the 10-min course and have overlaps with classes of iron homeostasis as well as metabolic classes (tricarboxylic acid, TCA) and electron transport chain (ETC), Table S3). Targets of ArcA and Cra are enriched both in upregulated and downregulated sets. Another global aerobic regulator, FNR, is only enriched in the downregulated gene set.

GO classes related to iron homeostasis and iron transport predominate the list of upregulated genes (Figure 4a, lines 7, 8, 11, 12, 14, 16, 18). The other GO classes are related to aerobic metabolism, especially the TCA cycle (line 5), and aerobic respiration (lines 10, 13, 15, and 17). Upregulation of these groups appears to initiate later compared to iron homeostasis genes (at 2 or 5 min). Furthermore, genes linked to the generation of polyamines are overrepresented, especially in the 5- and 10-min samples (line 6).

Significantly overrepresented GO classes among downregulated genes can only be detected in the 5- and 10-min samples (Figure 4b). Gene sets associated with anaerobic electron transport predominate here (lines 8, 10, and 11). These classes overlap with the regulon of NarL (line 4). Even though no nitrate was present in the growth medium (Table S1), cells seem to express nitrate respiration genes just in case. The metabolic group of fermentative genes is only overrepresented in the 5- and 10-min samples (line 7) and can, therefore, be assigned to late responding genes.

The GO classes “generation of precursor metabolites and energy” and “energy derivation by oxidation of organic compounds” contain both aerobic as well as anaerobic genes. The classes are overrepresented in both GO analyses (Figure 4a, lines 4 and 13; Figure 4b, lines 6 and 9).

### 3.2. Transcriptional Elongation Rates

**Figure 5:**
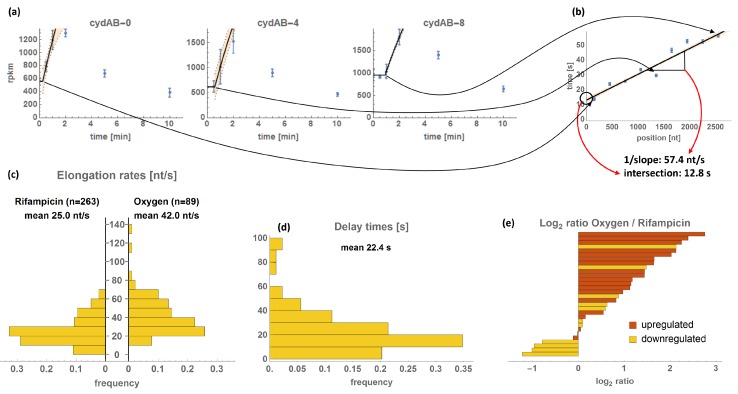
Calculation and overview of transcriptional elongation rates (TERs). (**a**) The calculation method is illustrated with the example of the *cydAB*-operon. Depicted is the expression of the 0th (1–300 nt), 4th (1201–1500 nt), and 8th (2401–2700 nt) bin of the operon (in RPKM ± standard deviation) together with the ordinary differential equation fit of the data. (**b**) The time points of the kinks are plotted over the positions of the respective bins. The inverse slope of a linear regression of these data gives the TER of the operon, the intersection with the *y*-axis (position 0 of the operon) gives the delay of transcription initiation. (**c**) Histogram of the resultant TERs compared to those obtained by Chen et al. [13]. (**d**) Histogram of the obtained transcription delay times. (**e**) Comparison of the TERs of individual operons obtained by oxygen and rifampicin methods; log_2_ ratios are indicated together with upregulation (red) or downregulation (orange) of the oxygen treatment group. Detailed data are available in Table S4.

TERs and transcriptional delay times of individual operons were determined by estimating the times at which expression of successive subsections of an operon begin to increase or decrease (Figure 5a,b). The average TER obtained does not account for possible fluctuations of the elongation speed. For readability, we will stick to the term TER instead of average TER throughout this article, bearing in mind that fluctuations may occur. Our expression data show both decreasing and increasing expression. In the former case, the rate of the last polymerase bound before the promoter became less active is determined. In the latter case, the rate of the first polymerase after promoter activation is tracked.

We were able to infer TERs for 89 operons with a median TER of 38.6 nt/s with the 10% quantile at 21.5 nt/s and the 90% quantile at 63.6 nt/s. The delay times of transcription initiation have a median of 17.9 s with the 10% and 90% quantiles at 4.0 s and 43.0 s, respectively (Figure 5c,d, Table S4). In few cases, negative delay times were estimated by linear regression and in one case (*adhE*) the 95% confidence interval does not include the zero. Those values are relatively small (up to −7 s) and can be assigned to variations of TERs within the operons (higher initial TER). In the histogram of delay times (provided in Figure 5d) the negative values have been changed to zero, indicating immediate expression.

#### 3.2.1. Correlation Analyses of TERs and Delay Times

What are the driving forces governing TERs? We approached this question by evaluating Spearman correlations of the obtained TERs with characteristics of the respective operons. Table 1 gives a summation of our findings (details available in Table S4). The CAI is a measure for the codon usage bias [38]. In our data, the estimated TERs negatively correlate with CAI (Table 1). The same holds for the more recent ITE [40]). All other correlations concerning expression levels, sequence features, sigma factors, and regulators were not significant (*p* value > 0.05).

Upregulation, as a binary feature, as well as high maximal FC, correlates positively with delay times. Contributions of the sigma factor S, and of the regulators Cra and FNR are correlated with short delay times. Conversely, the regulator Fur-Fe correlates with long delay times. Other regulators, as well as the number of regulators involved in the regulation of the respective operon, have no significant influence on the delay time (Table S4).

## 4. Discussion

### 4.1. Gene Expression Patterns

Cellular response to an aerobic shift can be characterized by early (0 to 1 min) and late (5 to 10 min) altered gene expression (Figure 3). Significant differential expression of 23 genes within 30 s (Table S2) indicates immediate cell adaptation to the changed environment. The early sampling points at 0.5 and 1 min enable the study of the order in which differential gene expression appears. As most of the dynamic changes take place within the initial 5 min after the switch, our dataset is much richer in detail compared to earlier studies [4,5] where the first sampling points are at 5 min and 2 min, respectively. The 2-min sampling point in Rolfe et al. [5] is sufficient to grasp also transient upregulation, such as in *cydAB*, which is missed in the Partridge et al. data [4]. However, the earlier time points are required to follow the increase rather than only the decrease in expression.

The total number of both upregulated and downregulated genes increases steadily during the initial 10 min after aerobic shift to 555 genes (Figure 1). However, the composition of DE genes is not just increasing, but rather changing with time. Besides early and late cell response, gene expression data reveal transient up- or downregulation (e.g., Figure 3f) as a common characteristic (Figure S2). Several reasons can be conceived as to why transient expression could be advantageous for the cell: (1) expression of stress genes, as the stress from oxygen is only present until the cell has adapted to it; (2) expression of enzymes that are optimal under microaerobic conditions. Microaerobic conditions occur right after the initiation of aeration, however, only for a few seconds, as 100% aerobiosis is reached at a pO_2_ between 0.5 and 5% when oxidases are saturated (Figure S1 [5,44]); (3) by temporarily overexpressing the transcript of a required enzyme, total translated amounts of the respective protein will be higher, i.e., in the competition for ribosomes, the more abundant mRNAs will be translated proportionally [45]; (4) transient need for alternative metabolic enzymes due to capacity constraints [8,46]. The capacity constraints arise from slow gene expression, or from inactivation of the preferred enzymes. Alternative enzymes are often enzymatically less efficient (i.e., more enzymatic transformations are needed for the same net reaction) or metabolically less efficient (i.e., less energy is conserved from the reaction; this classification refers to parsimonious enzyme usage flux balance analysis [12]). Not surprisingly, PCA detected the distinction of increasing or decreasing expression as the feature that accounts for most variance (Figure S3). The second property determined by PCA was early or late differential expression. This depends on the time needed for regulation and on the position of the gene within the operon (i.e., the distance from the promoter). In the regulon and GO enrichment analysis, the iron regulator Fur, together with GO classes iron transport and homeostasis (Figure 4a, lines 3, 7, and 16), turned out to be the earliest responding classes. In the following sections, we will discuss the expression of iron homeostasis genes and aerobic respiration genes in more detail.

#### 4.1.1. Iron Balance

The activity of many redox enzymes depends on the ability to oxidize ferrous iron (Fe^2+^) to ferric iron (Fe^3+^) in the presence of oxygen. Oxidation of Fe^2+^ poses stress on the cells through the inadvertent release of ROS in the Fenton reaction [47]. To encounter emerging ROS, cells maintain intracellular iron homeostasis by adapting iron uptake and storage [47,48]. Previous studies have suggested the emergence of a burst of ROS stress upon the transition to aerobic conditions which was deduced from the transient expression of stress genes, such as *ahpCF* (an OxyR target) or increased activity of the regulator OxyR [4,5]. Transient upregulation of *ahpCF* is also evident from our data (Table S2) which leads us to the reasonable assumption of oxidative stress. The GO class “response to oxidative stress”, containing 88 genes, is enriched in the 5- and 10-min samples, though there are indications that this stress is present earlier (e.g., *ahpCF* is upregulated starting at 1 min).

The cells react to the oxidative stress by fast expression of iron homeostatic genes (Figure 4). This is also evident from the differential expression of *bfd* which holds the highest FC at 30 s after the aerobic shift (Table S2). Bfd, an iron-sulfur (FeS) cluster containing ferredoxin, is involved in iron storage and reveals the importance of iron homeostasis caused by an aerobic shift [49,50].

At the same time, the aerobic shift induces expression of ETC genes, whose products require Fe^2+^ for their activity (Figure 2a–c and Figure 3c,e). In turn, this activity contributes to reducing oxidative stress by facilitating a controlled reduction of molecular oxygen [48]. Consequently, cells dynamically regulate iron homeostasis according to its demand.

#### 4.1.2. Expression of Oxidases

In response to an aerobic shift, *E. coli* adapts the expression of its respiratory enzymes cytochrome bo oxidase (encoded by *cyoABCD*, Figure 2a–c and Figure 3e) and cytochrome bd-I oxidase (encoded by *cydAB*, Figure 5a). Functional cytochrome bo oxidase contains ferrous iron bound to a heme group. The genes *cyoA* and *cyoD* were identified by PCA as representatives for early and late upregulation (Figure 3c,e), respectively, which can be ascribed to the positions of the genes within the *cyo*-operon. In contrast to the *cyo*-operon, expression of *cyd*-genes is increased even earlier (13-s versus 17-s delay, Table S4) and is only transient. Expression of Cyd, the main oxidase under microaerobic conditions, is potentially triggered by the short microaerobic phase after onset of aeration [51]. Considering its high O_2_ affinity, the oxygen-scavenging enzyme Cyd facilitates O_2_ sensing of the ArcBA two-component system by oxidizing the quinol pool even in microaerobiosis [48,52,53,54]. Cyd is less efficient in terms of energy conservation than Cyo. Hence, low expression levels of Cyd and high levels of Cyo under long-term aerobic conditions are beneficial for the cell. During the aerobic shift, both oxidases cooperatively reduce oxidative stress and contribute to the dynamic adaptation to the changed environment.

### 4.2. Transcriptional Elongation Rates

Using oxygen, a naturally occurring stimulus, essential transcriptional dynamics of the cellular response were investigated on a short time scale. Our obtained TER values are in good agreement with earlier reports that used pulse chase or hybridization techniques for TER estimation [55,56]. We compared our TER results to Chen et al. [13], who used rifampicin to block transcription initiation. Rifampicin binds free RNA polymerases irreversibly and globally prevents transcription initiation [57]. 30 of our analyzed operons are contained in the results of Chen et al. (Figure 5e).

In general, the mean TER was higher after oxygen treatment than after rifampicin treatment (Figure 5c). The ratios of TER_oxygen_/TER_rifampicin_ range from halved rate to no difference to 7-fold increase (Figure 5e). High TER_oxygen_/TER_rifampicin_ ratios are correlated with an upregulation in the oxygen treatment group (while after rifampicin treatment, all operons are downregulated). The difference between the measurement of upregulated and downregulated operons is that at downregulation, the operon sequence is initially loaded with RNA polymerases. Simplified, as soon as the promoter is turned off, the position of the endmost polymerase can be tracked. Conversely, at upregulation, the operon sequence is empty initially and the position of the first polymerase binding after the promoter is switched on can be followed. It has been shown that subsequent polymerases are able to push the preceding polymerase over roadblocks and prevent proofreading, thereby accelerating transcription [15,58]. This is not the case for the polymerase that is last in line, whose position is determined in operons with decreasing expression. This effect could explain the strong association of upregulated operons with increased TER compared to rifampicin (Figure 5e). The mean TER was higher in our study (42.0 nt/s) compared to Chen et al. (25.0 nt/s, Figure 5c). This might be a result of the high representation of upregulated operons (62 of 89).

Otherwise, upregulation does not generally correlate with TER, indicating that other factors are more pivotal for TER. In addition, the maximal FC or maximal RPKM, indicators for strong promoters, do not correlate with fast TERs, even though a high polymerase density is likely present (Table 1). We did not expect this, because in the case of upregulation, fast TERs might contribute to increase transcript levels and vice versa for downregulated operons. However, the opposite seems to be the case. The strong correlation between delay times and maximal FCs suggests that strong induction results in delayed polymerase binding. However, high FCs might also (at least in part) be attributed to differences in transcript stability rather than synthesis.

Concerning sequence features, the GC content, or any other nucleotide content (Table S4), was irrelevant for TER, even though more hairpin loops are formed in GC-rich sequences. This result is consistent with the finding of Proshkin et al. [15], that hairpins are not responsible for TER decrease. CAI is a measure of codon usage bias of a sequence and high CAI values are associated with fast translation rates [38,59,60,61]. Contrary to our data (Table S4), a positive correlation between CAI and TER was suggested, assuming cotranscriptional interactions between ribosomes and RNA polymerases [15,16]. We found a significant negative correlation between CAI and TER which argues against the stimulation of TER by ribosomes during cotranscriptional translation. This means that common codons are transcribed slower than rare codons and contradicts previous findings made on a small scale [15]. The recently developed ITE, which is claimed by the author to be a superior indicator of translation elongation, also negatively correlates with TER (Table S4). We also checked for correlation of the RNA folding energy with TER. The underlying idea was that the folding of the nascent RNA chain pushes the polymerase forward. We found no significant effect.

The second feature obtained from TER estimation is the delay time from stimulus until transcription sets in. This delay depends on the time required for regulators to sense the stimulus and to set up differential regulation. By comparing our data with various regulators and sigma factors we identified Cra, FNR, and sigma S as regulators correlated with short delay times. FNR is involved in the regulation of aerobic and anaerobic enzymes [62]. It is directly inactivated by oxygen (via oxidation of a FeS cluster [63,64]). This explains the short delay. Cra is a global regulator of carbon metabolism and influences expression of genes related to gluconeogenesis, TCA cycle, and glycolysis, among others [65]. Effectors of Cra are fructose-1-phosphate and fructose-1,6-bisphosphate which act as sensors of glycolytic flux (which is, in turn, higher in anaerobic cultures than under aerobic conditions [66,67]). Compared to the redox state, this effect is rather indirect. We therefore expected changes of fructose-phosphate levels to occur later than changes in the redox state of the cell, so that FNR-regulated operons were expected to have shorter delays than Cra regulated operons, which was not the case in our data. Sigma S is the sigma factor responsible for general stress response [68]. For this reason, we expected a fast response of sigma S-dependent operons, which is confirmed by our data.

Genes regulated by the ferric-uptake regulator Fur-Fe are correlated with long delay times. Fur-Fe targets are mainly involved in iron homeostasis [69] and its targets were found to be enriched from the 0.5 min sample on. Fur binds to its DNA target sequences in the presence of intracellular ferrous iron (Fe^2+^) thereby repressing expression of target genes. The redox potential of intracellular iron is much higher than that of iron bound to FeS clusters (FNR) or cysteines in a disulfide bond (OxyR) [70,71]. This can cause the long delay time of Fur-Fe depending operons. Furthermore, an upregulation of *fur* can be observed after the stimulus (Table S2). Varghese et al. show that upregulation of the regulator Fur through OxyR in an oxidative stress environment counteracts the inactivation via oxidation and restores repression of Fur target genes [72,73]. This mechanism prevents the early import of iron which would result in additional ROS stress through the Fenton reaction.

However, Fur targets overlap to a large extent (*p* value of the Spearman correlation of 0.002, Table S4) with the feature upregulation which correlates with long delays. Ultimately, this might be the underlying cause for the longer delay of Fur targets. Cra and FNR targets correlate insignificantly with downregulation which might contribute to their observed fast initiation.

Concerning expression levels, upregulation and maximal FC correlated positively with the delay times, indicating that the cell response via upregulation takes longer than via downregulation. This effect might be assigned to the competition of promoters to RNA polymerases [74]. Since more genes are upregulated than downregulated in our experiment, more active promoters compete for the same number of polymerases and recruitment takes longer. Formation of an active transcription complex is a multistep process and several time constants apply to get from the closed initiation complex to the active open complex conformation [75]. This additional time is not required for stopping transcription (here, only binding or dissociation of the respective regulators are needed). The faster response of downregulated operons is, therefore, plausible. The two properties “upregulation” and “maximal FC” resemble each other (low values or zero for downregulation, high values or 1 for upregulation). Therefore, similar correlations were expected.

The range of total transcription times (from stimulus until completed transcription) from 30 s to 5 min is similar to the mixing times of large-scale bioreactors. An average cell travels in one mixing time through the reactor volume from an oxygen-rich to an oxygen-depleted environment and back. This means that in most cases the stimulus (here oxygen) will be gone before even transcription of an operon is finished. Yet more time is needed for translation and post-translational modifications, so the point where the response to the stimulus is completed becomes independent from the actual environment. This needs to be investigated, e.g., by applying continuing shifts in aeration to a laboratory scale culture.

The *ndh* operon, containing only one gene, is the fastest among upregulated operons with 34 s (Table S4, Figure 3f). The iron-independent Ndh catalyzes the first step in the ETC but it is metabolically less efficient than Nuo [76,77]. Ready availability, as demonstrated by the short total transcription time, is an advantage of *ndh* and might be a reason for its upregulation (Figure 3f [8]). Expression of less efficient genes might be in part responsible for yield losses observed in large scale fermentations [3,78].

## Figures and Tables

**Table 1:** Spearman correlations (*ρ*) of TERs and delay times with expression levels, sequence features, regulators, and sigma factors.

	*ρ* of TER	*p* Value	*ρ* of Delay Time	*p* Value
upregulation	−0.084	0.43	0.37	**2.7 × 10^−4^**
maximal FC	−0.17	0.10	0.416	**7.2 × 10^−6^**
maximal RPKM	−0.10	0.36	0.05	0.61
GC content	−0.11	0.32	−0.04	0.73
CAI	−0.23	**0.03**	−0.10	0.35
ITE	−0.25	**0.02**	−0.15	0.14
RNA folding energy	0.17	0.10	0.12	0.25
regulated by Cra *	−0.03	0.75	−0.29	**0.01**
regulated by FNR *	−0.13	0.21	−0.21	**0.04**
regulated by Fur-Fe *	−0.12	0.28	0.22	**0.04**
controlled by sigma S	−0.15	0.15	−0.22	**0.04**

* without differentiation between activation or repression. Values in bold indicate significant correlation (*p* value < 0.05). CAI: codon adaptation index; FC: fold change; ITE: index of translation elongation; RPKM: reads per kilobase per million reads; TER: transcription elongation rate.

## Supplementary Materials

The following are available online at www.mdpi.com/2073-4425/8/3/90/s1. Figure S1: Dissolved oxygen concentration increases after the onset of aeration. Figure S2: Heatmap of logFCs of DE genes. Genes with differential expression in at least one time point were analyzed for transient expression patterns and plotted in the order of the FC at 10 min compared to anaerobic control. (1 and 2) Genes with increased expression after 10 min; (1) without transient upregulation; (2) with transient upregulation; (3 and 4) genes with decreased expression after 10 min; with (3) and without (4) transient downregulation. The sections 2 and 3 indicate genes with transient effects and make up about half of all DE genes. Figure S3: Scree plot of PCA. The plot shows the percentage of variance of the principal components. Figure S4: Rotation of PCA. The rotation plot indicates the contributions of all genes to the PCs 1 and 2. Gene names can be identified by pointing at the dots in the plot. Figure S5: Regulon and GO enrichments of upregulated (a) and downregulated (b) genes compared to anaerobic state. *p* values with Bonferroni correction of the enrichments are represented. Table S1: Composition of defined medium, modified from [79]. Table S2: Differential expression: logFCs are provided together with expression levels and *p* values for differential expression for all genes analyzed. Table S3: Regulon and GO enrichment analyses. *p* values and matched genes for every class are indicated. Table S4: TERs and delay times of analyzed operons. Pearson and Spearman correlations with respective *p* values are provided for TER and delay times after aerobic shift.
