# Supplementary material for: Rapid Sampling of Escherichia coli After Changing Oxygen Conditions Reveals Transcriptional Dynamics
Source: Genes (Basel). 2017 Feb 28;8(3):90. doi: 10.3390/genes8030090 (PMC5368694; doi:10.3390/genes8030090)
Supplement: Supplementary file 1 [file genes-08-00090-s001.zip › supplementary files/addFig4_rotation.html]

xml version="1.0" encoding="UTF-8"?


Additional Figure 4: Principal Component Analysis


Created with the Wolfram Language
