# Supplementary figures and images for: Rapid Sampling of Escherichia coli After Changing Oxygen Conditions Reveals Transcriptional Dynamics
Source: Genes (Basel). 2017 Feb 28;8(3):90. doi: 10.3390/genes8030090 (PMC5368694; doi:10.3390/genes8030090)

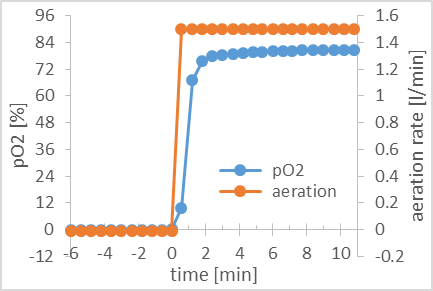

Supplement: Supplementary file 1 [file genes-08-00090-s001.zip › supplementary files/addFig1_DO.png]

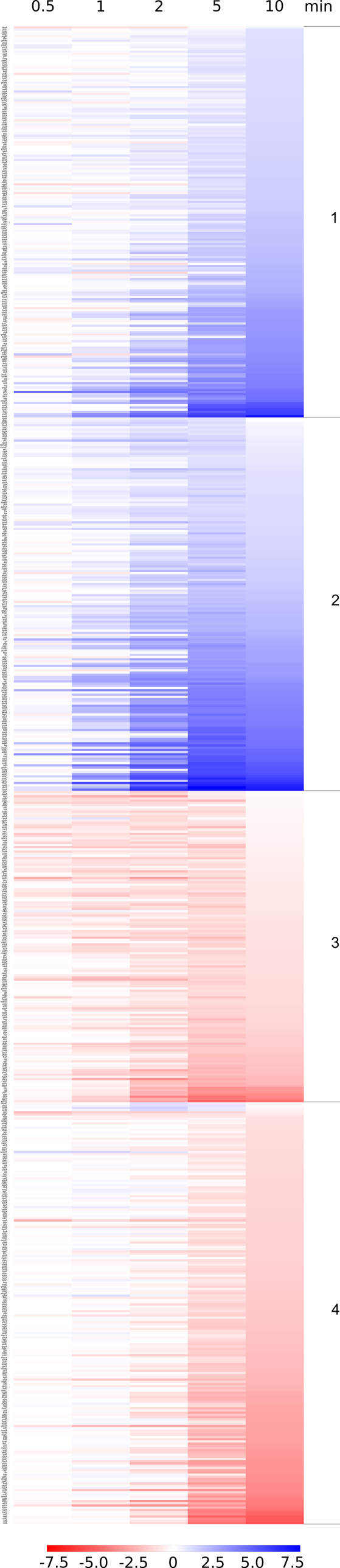

Supplement: Supplementary file 1 [file genes-08-00090-s001.zip › supplementary files/addFig2_heatmap.pdf]

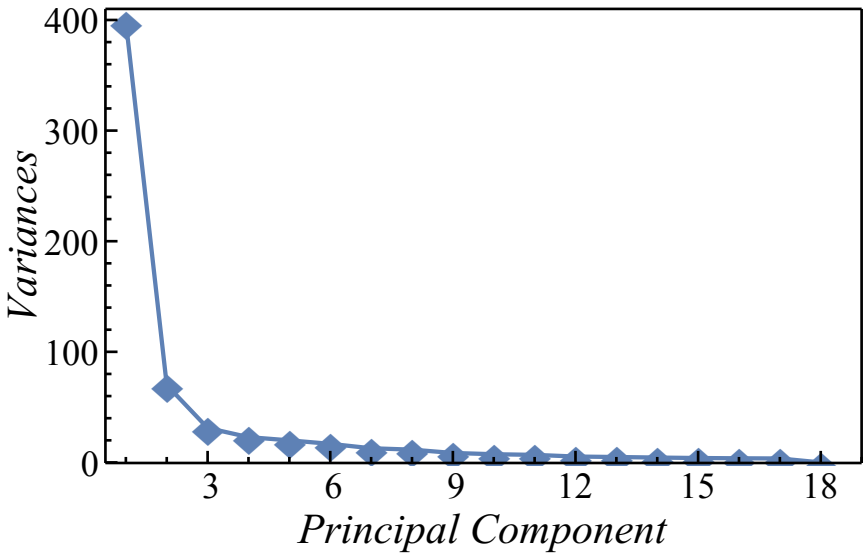

Supplement: Supplementary file 1 [file genes-08-00090-s001.zip › supplementary files/addFig3_screeplot.pdf]

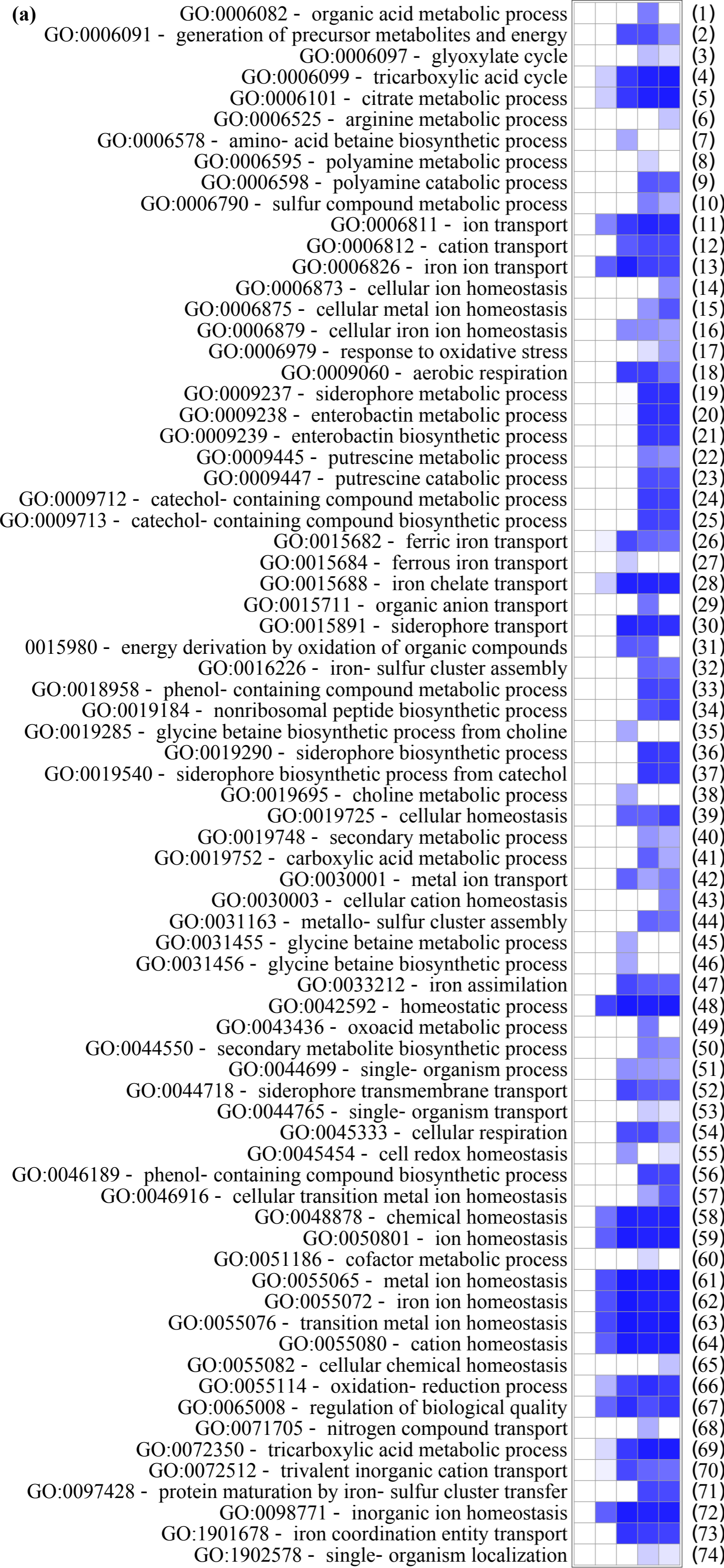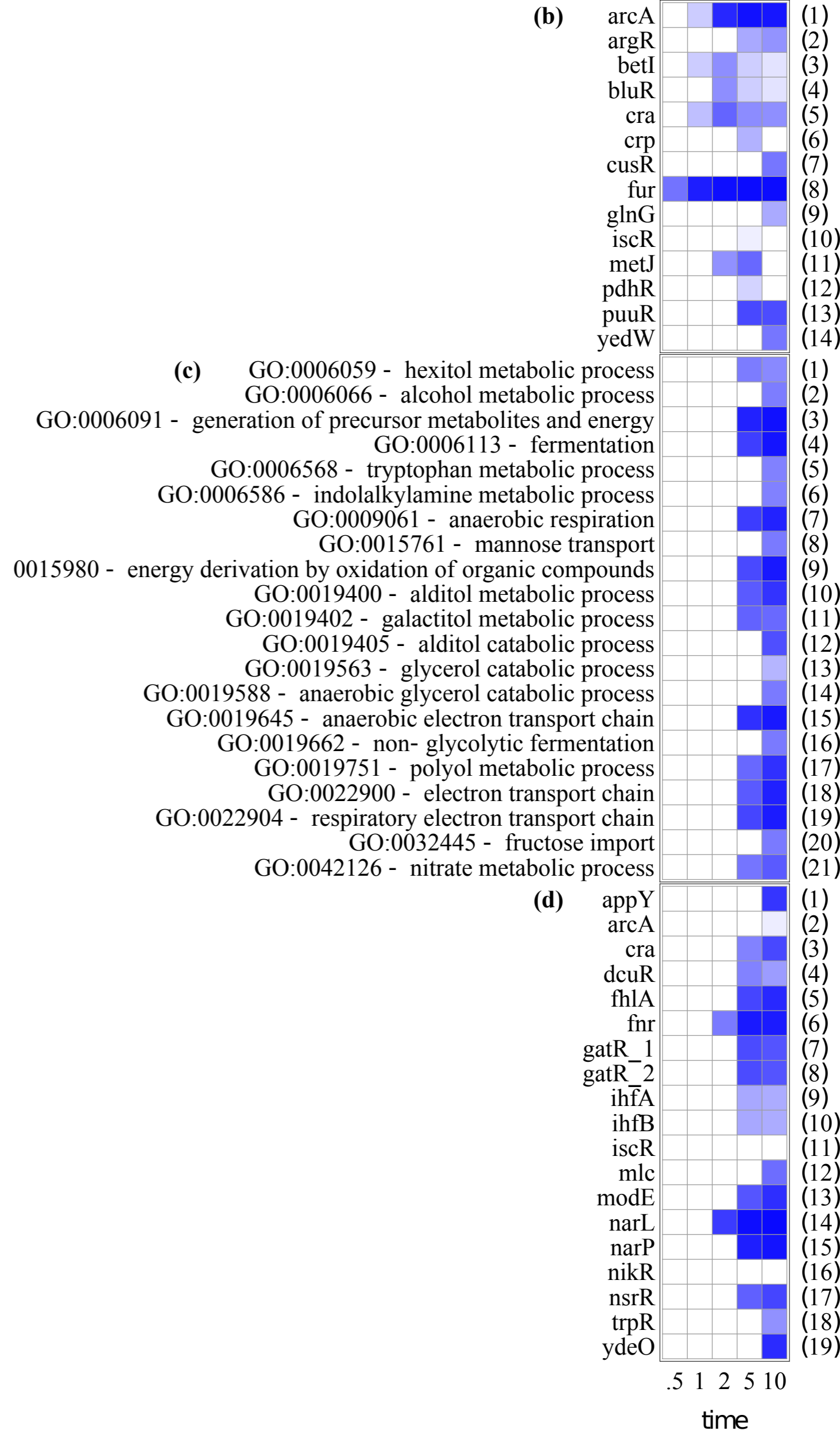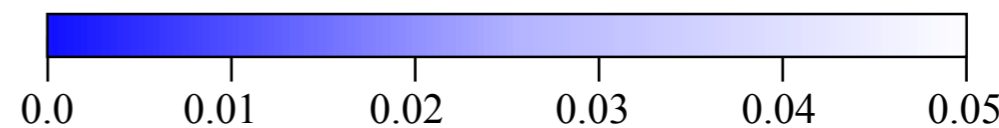

Supplement: Supplementary file 1 [file genes-08-00090-s001.zip › supplementary files/AddFig5_enrichments.pdf]

(a)

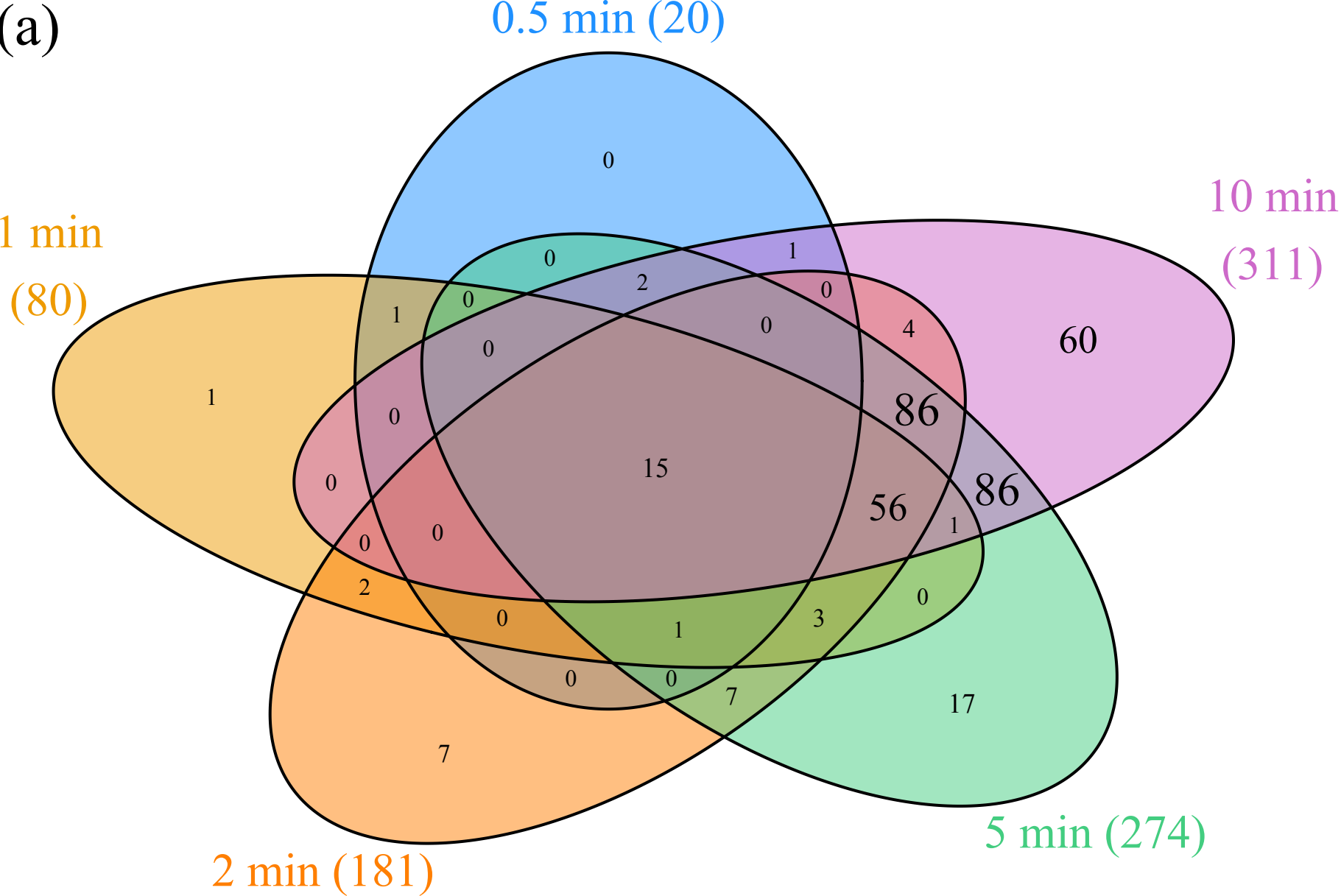

(b)

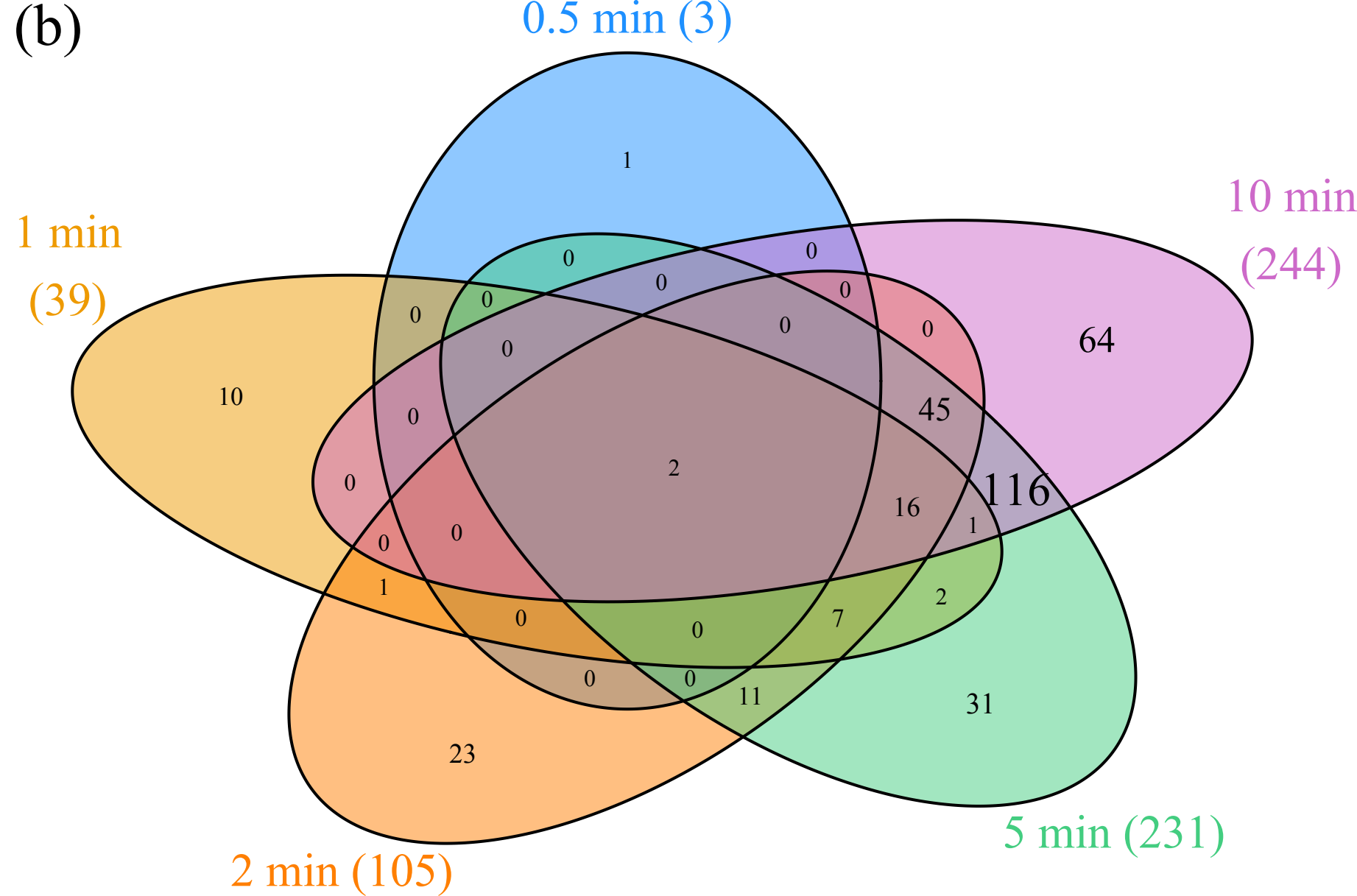

Supplement: Supplementary file 1 [file genes-08-00090-s001.zip › supplementary files/Fig1_Venn.pdf]

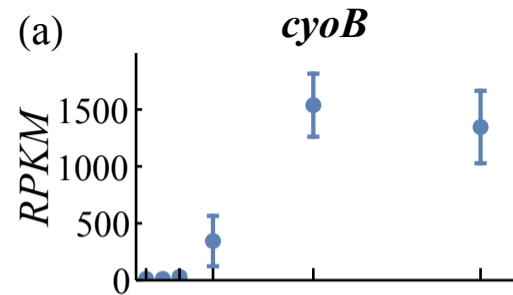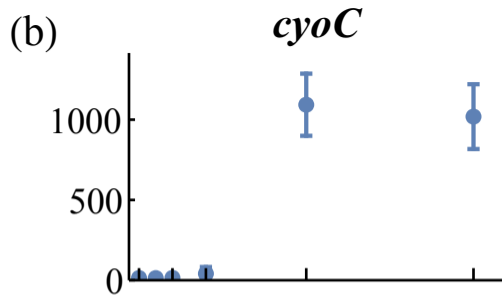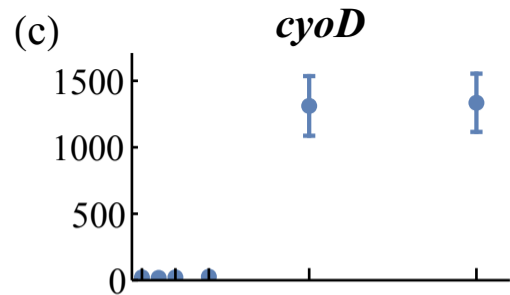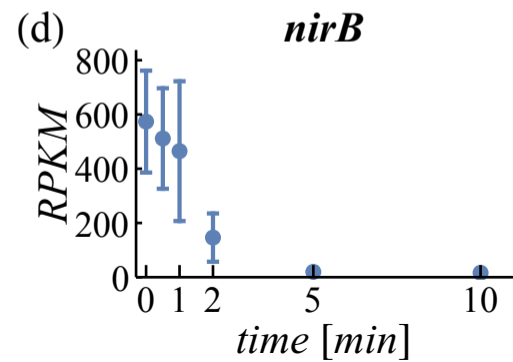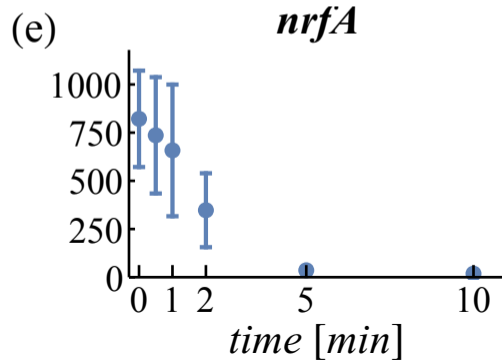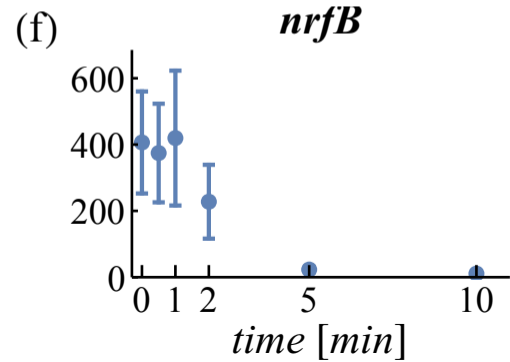

Supplement: Supplementary file 1 [file genes-08-00090-s001.zip › supplementary files/Fig2_PC1loading.pdf]

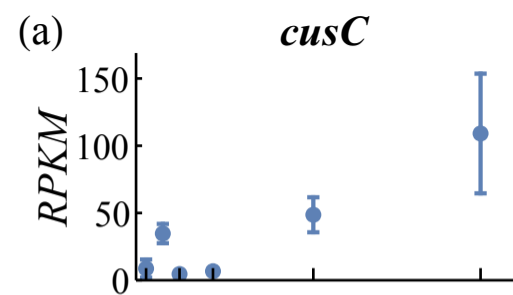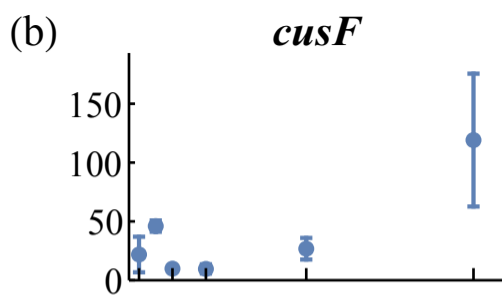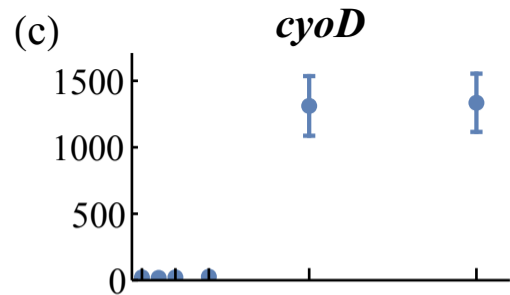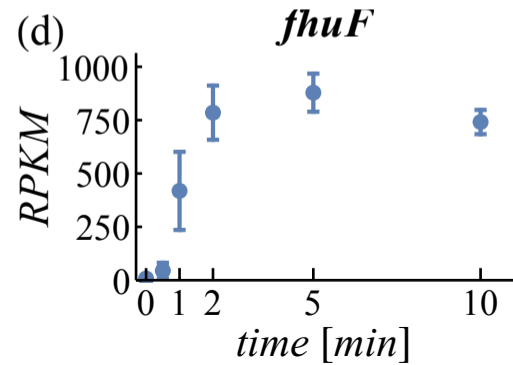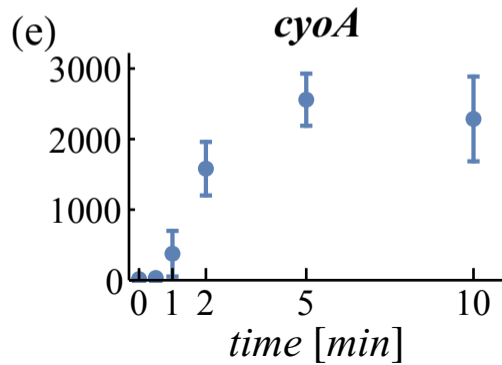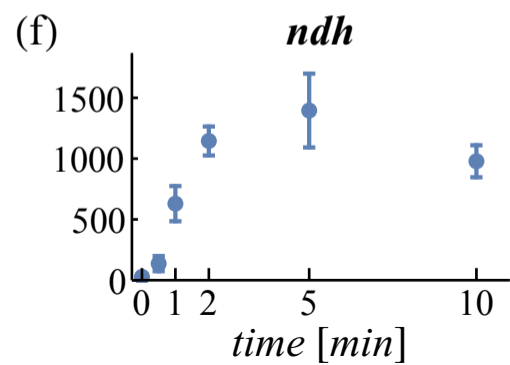

Supplement: Supplementary file 1 [file genes-08-00090-s001.zip › supplementary files/Fig3_PC2loading.pdf]

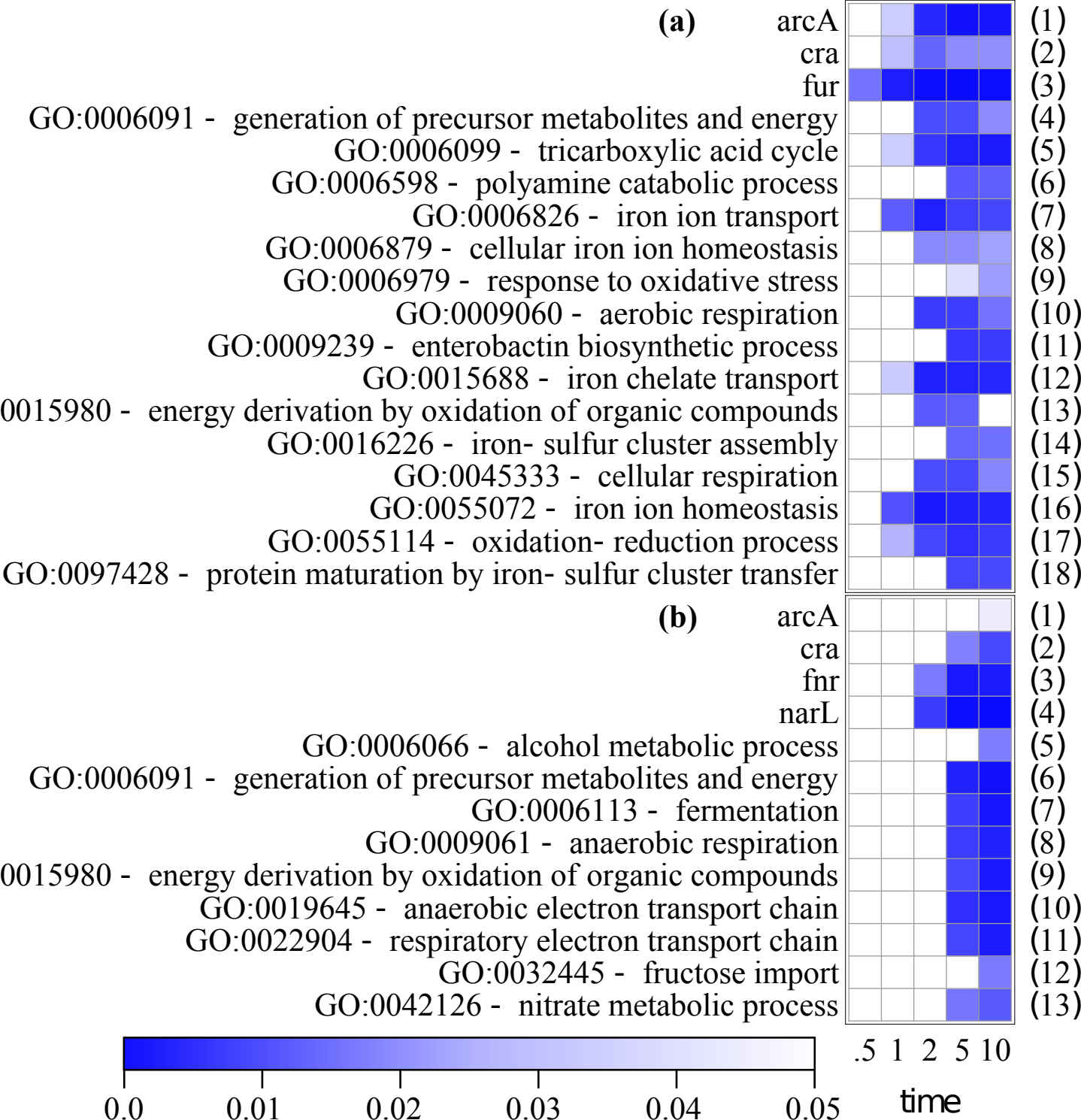

Supplement: Supplementary file 1 [file genes-08-00090-s001.zip › supplementary files/Fig4_enrichments.pdf]

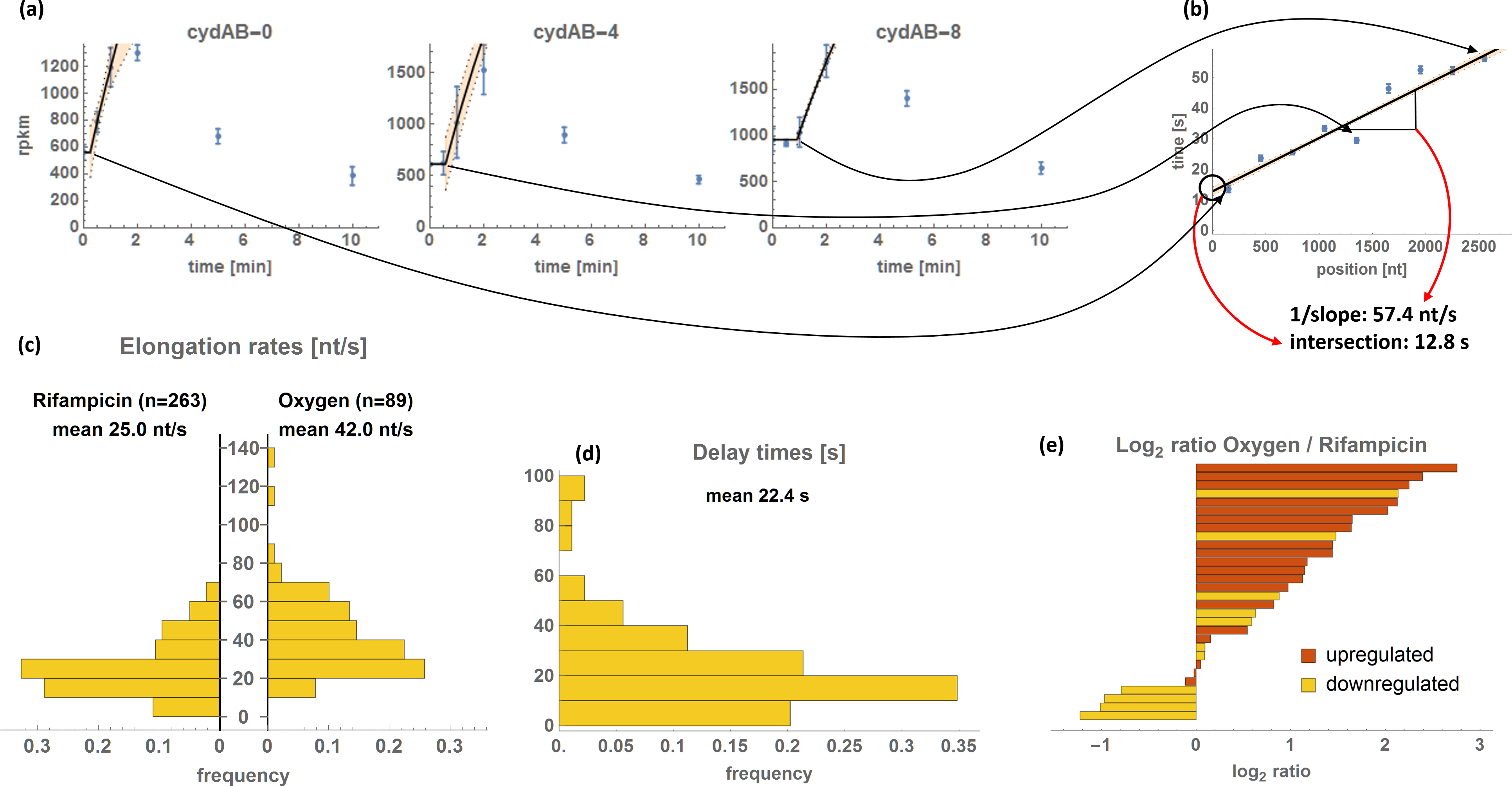

Supplement: Supplementary file 1 [file genes-08-00090-s001.zip › supplementary files/Fig5_TER.png]
